# Case Report: A hypothesis-generating case of gadolinium retention and persistent symptoms after MRI despite normal renal function in a patient with hypermobile Ehlers-Danlos syndrome

**DOI:** 10.3389/ftox.2026.1846464

**Published:** 2026-07-16

**Authors:** Cassandra Cox, Abigail Unruh, Brent Wagner, G. Patricia Escobar, Catriona Walsh, Olivia X. Jastrzemski, Ian Henderson, Joshua DeAguero

**Affiliations:** 1 Kidney Institute of New Mexico, Internal Medicine, University of New Mexico Health Science Center, Albuquerque, NM, United States; 2 New Mexico Veterans Affairs Healthcare System, Associate Chief of Staff Research Section, Albuquerque, NM, United States; 3 Catriona Walsh, The Food Phoenix, Maghera, United Kingdom; 4 University of New Mexico Health Science Center, University of New Mexico, Albuquerque, NM, United States

**Keywords:** Ehlers-Danlos syndrome, gadolinium retention, gadolinium-associated symptoms, gadolinium-based contrast agents, genetic variants, magnetic resonance imaging, nanoparticles, oxidative stress

## Abstract

Gadolinium-based contrast agents are commonly used to enhance magnetic resonance imaging. In 2006, Thomas Grobner identified a link between these agents and nephrogenic systemic fibrosis in patients with renal impairment. Gadolinium exposure has also been associated with acute kidney injury, skin plaques, and encephalopathy. Even in patients with normal kidney function, gadolinium may be retained in tissues for prolonged periods after contrast administration. Some patients develop persistent and varied symptoms following exposure. Although Richard Semelka and colleagues proposed the term gadolinium deposition disease, uncertainty regarding causality and pathophysiology has led to the growing adoption of the more neutral description: symptoms associated with gadolinium exposure. We describe a patient with normal renal function and Ehlos-Danlos Syndrome who developed persistent symptoms following a single gadopiclenol-enhanced magnetic resonance imaging study. Serial testing demonstrated prolonged urinary gadolinium excretion despite normal kidney function and an undetectable blood gadolinium concentration. Gadolinium remained detectable in the urine long after the expected period of elimination. The patient’s clinical manifestations persisted for an extended period following exposure. We hypothesize that slow redistribution of gadolinium from retained tissue stores contributed to the prolonged urinary excretion observed in this patient. We also discuss theoretical host-susceptibility factors that may have increased her risk of gadolinium retention following gadolinium-enhanced magnetic resonance imaging. However, this case adds to the ongoing discussion regarding the clinical significance and potential mechanisms of persistent gadolinium retention in patients with normal renal function.

## Introduction

Gadolinium-based contrast agents (GBCAs) are widely used to enhance magnetic resonance imaging (MRI). Although generally considered safe, gadolinium exposure has been associated with nephrogenic systemic fibrosis (NSF) in patients with severe renal impairment, and gadolinium retention has been documented in multiple tissues even among individuals with normal kidney function (Cowper et al., 2001; Grobner and Prischl, 2007; Marckmann et al., 2006; Erley et al., 2004; Cowper, 2003; Moradian et al., 2022; McDonald et al., 2015; Kanda et al., 2014; Vymazal and Rulseh, 2024; Darrah et al., 2009). Some patients report persistent symptoms following GBCA exposure despite normal renal function. Semelka and colleagues proposed the term gadolinium deposition disease (GDD) to describe this clinical presentation. In contrast, the American College of Radiology has adopted the more neutral term Symptoms Associated with Gadolinium Exposure (SAGE), as causality remains uncertain (Darrah et al., 2009; Burke et al., 2016; Semelka et al., 2016; McDonald et al., 2022) (Table 1).

**TABLE 1:** Comparison of GDD diagnostic criteria and SAGE symptoms associated with gadolinium exposure.

GDD – Diagnostic criteria (Semelka et al.)	SAGE - Symptoms associated with gadolinium exposure (ACR)*
Normal renal function (not in severe kidney disease, which is NSF risk) Exposure to a gadolinium-based contrast agent (GBCA) Onset of symptoms within hours to 1 month after GBCA exposure Persistence of symptoms for >3 months Presence of ≥3 hallmark symptom clusters – Burning/prickling in skin8** – Pain in bone, joints, tendons, or ligaments** – Brain fog/cognitive complaints** – Headache** – Muscle fasciculations/twitches** – Skin thickening/discoloration8 Symptoms are new (not pre-existing or explained by another condition) No other diagnosis that better explains the constellation of findings	Pain (deep bone pain, joint pain, muscle pain)** Headache** Cognitive difficulties (“brain fog”)** Peripheral neuropathy (burning, tingling, pins-and-needles)** Skin thickening or discoloration** Muscle fasciculations, twitching, cramps Fatigue Dermal plaques, skin tightening** Other nonspecific systemic symptoms (reported in literature, not fully validated)

* Examples of reported symptoms.

** Persistent symptoms experienced by our patient reported within a month of exposure to gadolinium.

The clinical significance of prolonged gadolinium retention is debated, and the potential mechanisms underlying persistent symptoms reported after gadolinium exposure remain under investigation (McDonald et al., 2022; McDonald et al., 2018; Cunningham et al., 2024; Domingo and Semelka, 2025; Coimbra et al., 2024). Here, we describe a patient with persistent symptoms and prolonged urinary gadolinium excretion following contrast-enhanced MRI despite normal kidney function. Although our patient met the published diagnostic criteria for proposed gadolinium deposition disease (GDD), GDD has not been universally accepted or validated as a distinct disease entity. We also discuss potential susceptibility factors that may have influenced her clinical course.

## Case presentation

### Patient information

We present a case of a 37-year-old Caucasian woman of north/central European origin who received a 10 mL (0.5 mmol/mL) injection of gadopiclenol, a macrocyclic agent, into the right hand for MRI. She had a prolactin level of 51.4 ng/mL (normal range in a non-pregnant female: 4.8–23.3 ng/mL). Pre-MRI eGFR was 100 mL/min/1.73 m^2^. Historical urinalyses were unremarkable. The MRI revealed no abnormalities, and prolactin levels returned to normal values (17.5 ng/mL).

Our patient’s past medical history is significant for hypercholesterolemia, long-standing joint flexibility, knee dislocations, and congenital hip dysplasia. Early in life, she had tympanostomy tube placement, as well as a tonsillectomy and adenoidectomy. Family history includes hypertension and macular degeneration. Our patient is married and does not consume excessive alcohol or use social drugs. She is employed in technology and exercises regularly, both of which she continued to pursue to the best of her ability throughout her illness.

Her pre-MRI annual examination was unremarkable. Serum creatinine was 0.8 mg/dL, and eGFR was 97 mL/min. The only medications she was taking at that time were vitamin D and a multivitamin.

### Clinical findings

The patient quickly developed a cold arm, a metallic taste, a headache, and dizziness. Within hours, she developed abdominal pain, nausea, and severe diarrhea. These were followed by joint and limb pain.

**FIGURE 1:**
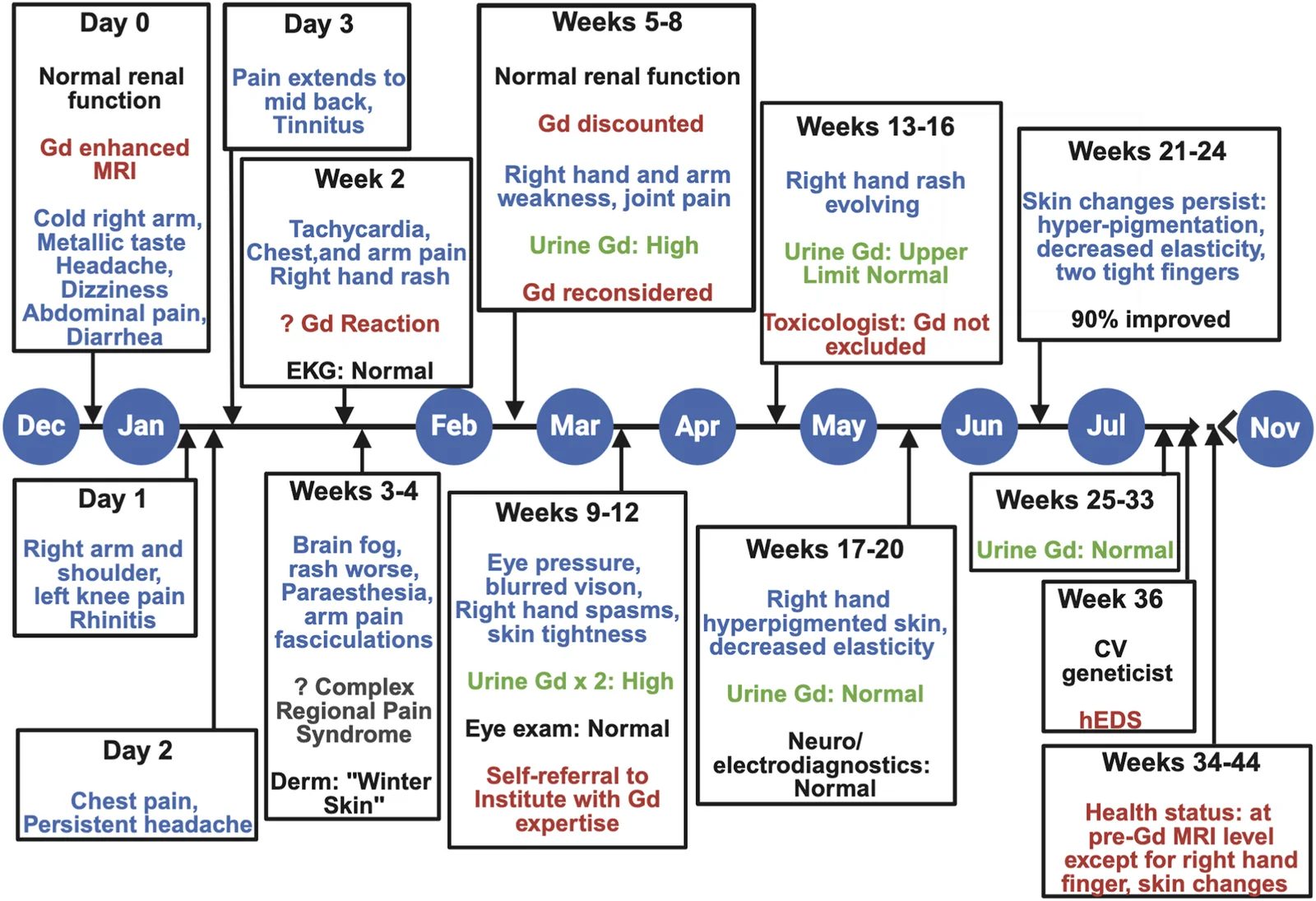
Timeline of the patient’s clinical course. Abbreviations: Gd, gadolinium; MRI, magnetic resonance imaging; EKG, electrocardiogram; CV, cardiovascular; hEDS, hypermobile Ehlers-Danlos syndrome. Created in BioRender. Cox, C. (2026).

Over the next few days, most initial symptoms related to the injection subsided. Arm pain worsened, and headache persisted, prompting an urgent medical evaluation by the on-call physician 9 days after the enhanced MRI. Physical examination was notable for diffuse tenderness to palpation of the right biceps and deltoid. She experienced pain with arm flexion past 120° and with resisted supination and pronation. Figure 1 presents a timeline of the patient’s clinical course.

Subsequently, the patient noted additional symptoms, including exertional tachycardia, migratory pain, neuromuscular disturbances, and skin changes that prompted multiple medical visits. Most symptoms developed within hours to days of exposure and persisted for months.

At follow-up 3 weeks after symptom onset, the patient complained of difficulty with concentration and memory, migratory pain involving the chest and arms, pins-and-needles sensations, and an icy sensation in her hand. She was alert with a heart rate of 109 beats per minute and blood pressure of 110/75 mmHg. She exhibited epicondyle tenderness and muscle fasciculations.

**FIGURE 2:**
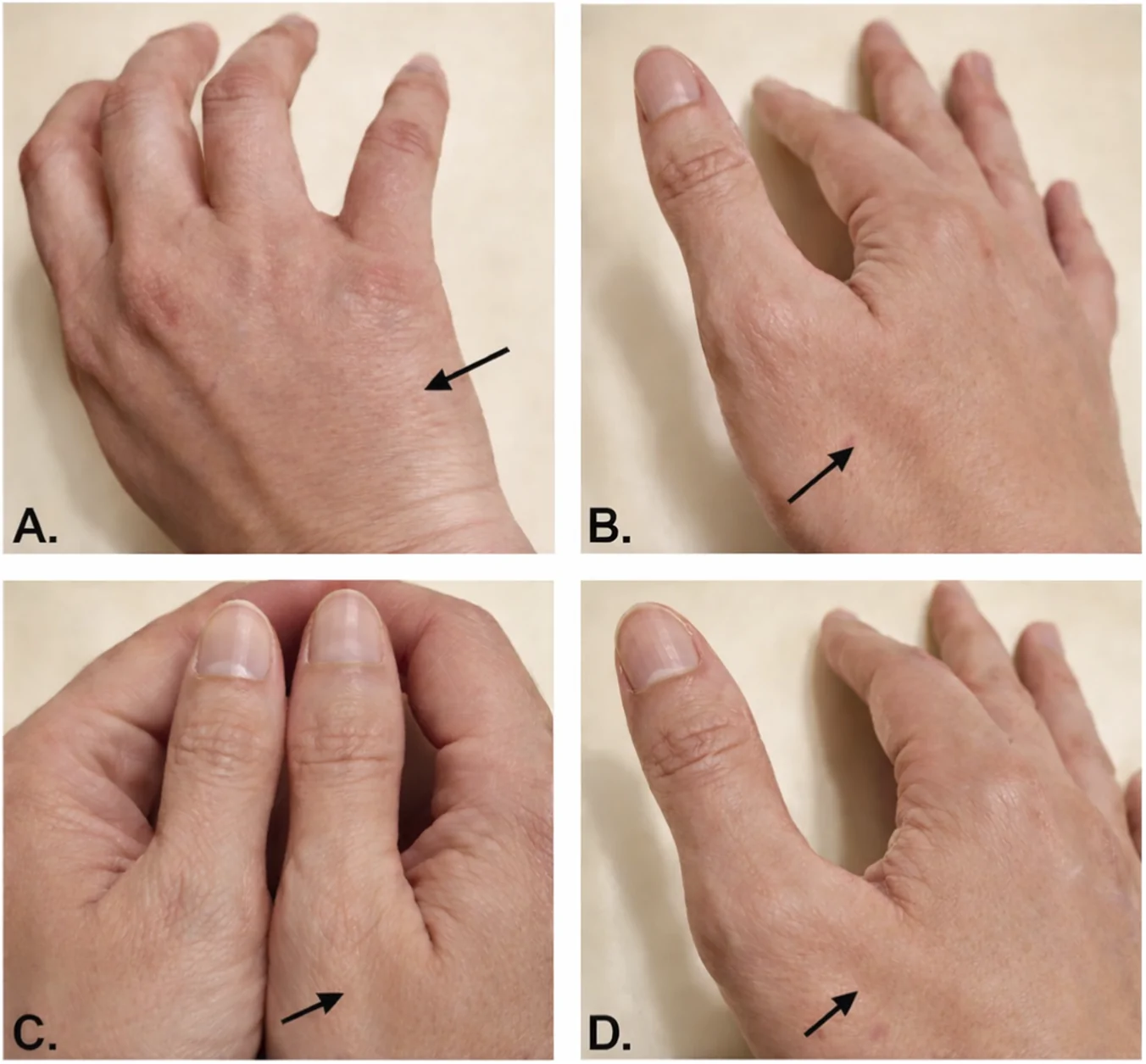
Right hand skin changes. Panels **(A,B)** show a diffuse erythematous rash on the dorsum of the right hand (arrows) that began 10 days after MRI with gadolinium and documented at day 22. Panels **(C,D)** show hyperpigmented areas with decreased elasticity on the dorsum of the right hand (arrows). Panels **(C,D)** are 224 days after MRI. Panel **(C)** includes the left hand for comparison.

Five weeks after the GBCA-enhanced MRI, the patient remained symptomatic, prompting her first visit with her primary care provider. Chest wall, elbow epicondyles, right knee fat pads, and shoulders were exquisitely tender. Very fine, pinpoint, raised pink lesions were noted on the proximal arms. Skin was dry, and the right hand had two flat areas of increased erythema. The patient documented progression of the rash on her right hand over time. Hyperpigmentation, tightness, and tingling involving the right ring and little fingers remain present (Figure 2).

Later in her clinical course, our patient experienced eye pain and eye pressure with blurred vision. Ophthalmic exam was normal.

### Diagnostics

The urgent evaluation included a right arm Doppler ultrasound to exclude thrombosis; the results were unremarkable. Although a nonallergic hypersensitivity reaction to gadolinium was initially considered, subsequent follow-up focused on the possibility of complex regional pain syndrome. However, as additional symptoms developed, including migratory pain, cutaneous changes, and systemic manifestations, the overall presentation was not readily explained by this diagnosis.

Noticing new symptoms and skin changes, along with symptoms temporally associated with the MRI within days, the patient suspected gadolinium as the cause and shared her concerns with her primary care physician. Given her preserved kidney function and the time elapsed since the exposure, her physician did not believe her symptoms were related to gadolinium exposure. Laboratory testing and a dermatology evaluation were ordered.

A comprehensive metabolic panel, complete blood count, sedimentation rate, thyroid function studies, coagulation studies, and vitamin B12 level revealed no abnormalities except an elevated cholesterol level. Serum creatinine was 0.7 mg/dL; cystatin C-based eGFR was 109 mL/min/1.73 m^2^. Dipstick urinalysis was unremarkable, with no evidence of proteinuria, hematuria, or other abnormalities suggestive of kidney injury. Albuminuria was not quantitated.

Early in her clinical course, the dermatologist diagnosed the hand rash as “winter skin,” a condition she had never previously experienced. The possibility of gadolinium-related toxicity was not entertained at that time. Given persistent symptoms and concern for a potential cardiac etiology, she underwent noninvasive cardiac testing, including electrocardiography, troponin measurement, and echocardiography, all of which were normal. Ambulatory cardiac monitoring demonstrated episodes of sinus tachycardia; however, these findings were not considered clinically significant.

Neurologic evaluation, including nerve conduction studies and electromyography, as well as vascular assessment, did not identify an explanation for her paresthesia, muscle tenderness, or fasciculations. Eye and visual complaints prompted ophthalmologic evaluation. Although an ocular migraine was considered, the patient had no prior history of migraine or similar symptoms.

**FIGURE 3:**
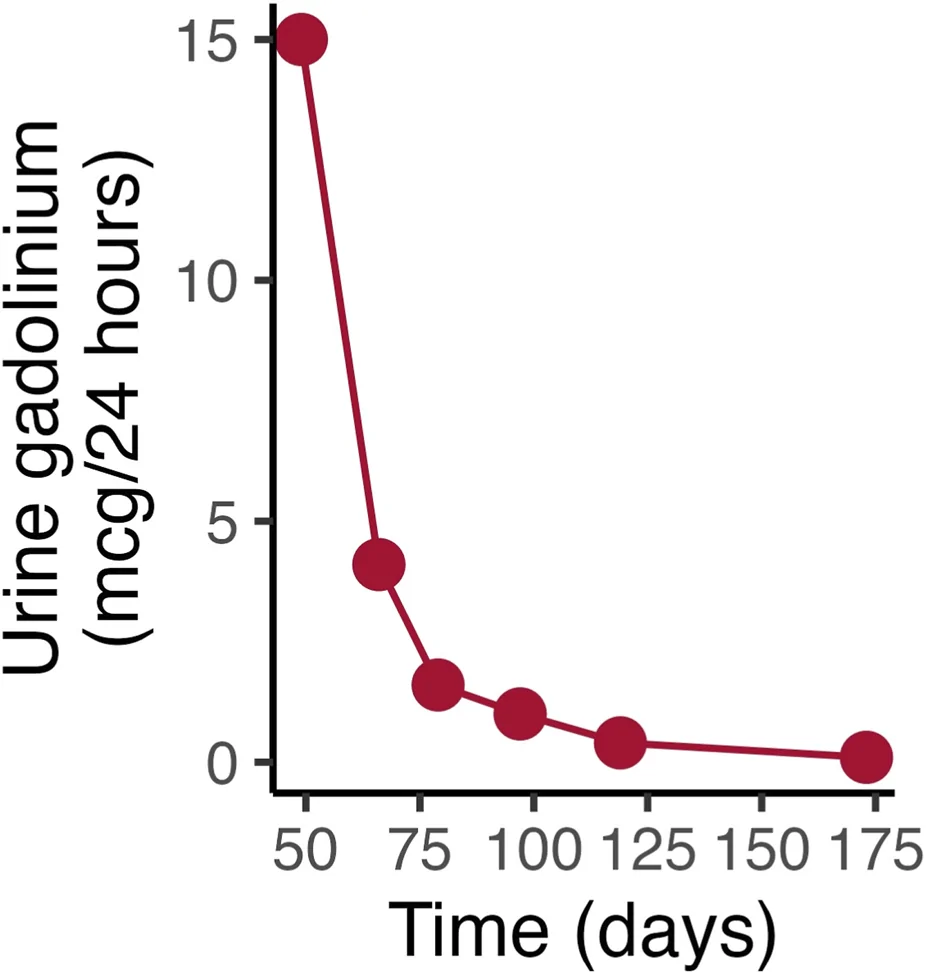
Serial 24-h urine gadolinium excretion measurements. Laboratory values were obtained through Quest Diagnostics/Mayo Clinic Laboratories (Rochester). Mayo Clinic Laboratories is a Clinical Laboratory Improvement Amendments (CLIA)-certified and College of American Pathologists (CAP)-accredited laboratory. The laboratory reference range for urine is <1.1 μg per 24 h. Created by R. Wagner, B (2025).

Unable to obtain a clear explanation, the patient independently researched gadolinium and convinced her provider to obtain additional testing. Serum and 24-h urine gadolinium measurements were performed by Mayo Clinic Laboratories using inductively coupled plasma mass spectrometry (ICP-MS). Serum gadolinium was not detected (reference value ≤ 0.4 ng/mL). Initial 24-h urinary gadolinium reported as total urinary gadolinium excretion (reference value < 1.1 µg/24 h) was elevated. Urinary gadolinium remained elevated for more than 3 months after MRI, indicating delayed excretion (Figure 3). Gadolinium was not above potable limits in the patient’s well water. With this information, she contacted our institute, a practice experienced with gadolinium toxicity research, and, at the advice of her primary provider, obtained a toxicology consult. The toxicologist could not exclude gadolinium as a contributor to her symptoms.

After most of her MRI-associated symptoms had resolved, the patient underwent evaluation by a physician specializing in genetic cardiovascular disease and was subsequently diagnosed with hypermobile Ehlers-Danlos syndrome (hEDS) using the 2017 International Classification criteria. The diagnosis was based on long-standing clinical features that predated gadolinium exposure (Malfait et al., 2017).

### Therapeutic intervention

Following provider guidance, the patient began physical therapy and hydration. Other self-directed treatments included red-light therapy, acupuncture, massage, sauna therapy, and vitamins and supplements based on commercial functional genetic testing. Clinicians discussed the lack of evidence supporting supplement use; however, she continued these interventions as part of what she considered a gadolinium detoxification strategy. She was counseled regarding the limited understanding of the potential risks associated with additional exposure to gadolinium-based contrast agents.

### Follow-up and outcomes

Serial testing demonstrated a progressive decline in urinary gadolinium excretion, eventually normalizing (Figure 3). Concurrently, symptoms gradually improved. Physical therapy, acupuncture, red-light therapy, and sauna therapy provided temporary symptomatic relief and were discontinued approximately 6 months into the illness. The patient perceived benefit from the supplements she initiated, although the extent of their contribution to recovery is uncertain. Massage therapy has been maintained for overall wellbeing. At the most recent follow-up, her health had largely returned to its pre-exposure baseline; however, hyperpigmentation, decreased skin elasticity, and intermittent right-hand tightness and paresthesia remain.

The subsequent diagnosis of hEDS provides a potential explanation for some musculoskeletal findings; however, the abrupt onset of new symptoms following gadolinium exposure, their gradual improvement over time, and the associated cutaneous changes are not readily explained by hEDS alone. Because connective tissue disorders have been observed in reported cases, we consider hEDS a potential susceptibility factor for her clinical course after receiving a gadolinium-based contrast agent rather than a unifying explanation for the patient’s entire presentation.

This patient’s presentation meets the proposed criteria for GDD. Although causality cannot be inferred, extensive evaluation did not identify an alternative explanation for our patient’s presentation.

### Patient perspective

My journey started with a routine MRI with contrast. I was not informed that contrast agents contain gadolinium or warned about potential side effects. During the scan, I experienced problems immediately after the injection and developed worsening symptoms in the following hours and days. Despite my concerns, physicians considered gadolinium unlikely due to my normal kidney function and first exposure.

As symptoms persisted and became debilitating, they took a significant physical, emotional, and financial toll. Navigating this largely alone, I advocated for testing and sought answers through my own research. This eventually connected me with researchers studying the effects of gadolinium exposure.

Further investigation, including genetic testing, suggested possible differences in detoxification and metal-handling pathways. I used this information to guide dietary and supplement changes during my recovery.

My experience illustrates the importance of informed consent for gadolinium administration-patients cannot recognize or report side effects if they are never warned about them. I hope my case highlights the challenges faced by individuals experiencing persistent symptoms after gadolinium exposure, particularly when the prognosis and management strategies remain incompletely understood. More research is needed!

### Discussion

In our patient with a diagnosis of hEDS and normal kidney function, a single exposure to a macrocyclic gadolinium-based contrast agent was temporally associated with self-described brain fog, muscle tenderness and fasciculations, periarticular tenderness, migratory pains, and visual disturbances. Additionally, documented skin changes and prolonged urinary gadolinium excretion occurred.

Urinary gadolinium concentration reflects cumulative renal elimination over time. In contrast, blood measurements represent only the circulating gadolinium level at the time of sampling and may be below the assay’s limit of detection despite measurable urinary gadolinium. Although persistent urinary excretion implies continued clearance of retained gadolinium, it does not prove tissue deposition, toxicity, or a causal relationship. However, these findings associated with gadolinium exposure justify further investigation.

A causal relationship between gadolinium exposure and nephrogenic systemic fibrosis (NSF) is widely accepted; the same is not true for GDD/SAGE. The underlying mechanisms for NSF are incompletely understood (Domingo and Semelka, 2025; Coimbra et al., 2024). However, the occurrence of NSF demonstrates that gadolinium-based contrast agents are not biologically inert. Still, the fact that only some exposed patients develop NSF suggests the presence of other host-specific factors (Rydahl et al., 2008; Deo et al., 2007). Regulatory actions in 2007 by the United States Food and Drug Administration (FDA) and the European Medicines Agency have reduced the risk of NSF, yet rare cases continue to be reported (U.S. Food and Drug Administration, 2026; European Medicines Agency, 2026). Subsequent studies have demonstrated gadolinium retention in the brain, skin, bone, and other tissues, prompting further regulatory action in 2017. However, the mechanisms responsible for tissue retention and their long-term clinical significance remain uncertain (McDonald et al., 2015; Kanda et al., 2014; Vymazal and Rulseh, 2024; Darrah et al., 2009; McDonald et al., 2018; U.S. Food and Drug Administration, 2017; European Medicines Agency, 2017).

Early on, investigators proposed simplified mechanisms of gadolinium toxicity, including transmetallation with the release of free gadolinium from the chelate (Idée et al., 2006). Transmetallation lacks direct *in vivo* evidence. Furthermore, physiological concentrations of endogenous cations are generally insufficient to displace gadolinium from highly stable macrocyclic chelates, and experimental studies have failed to demonstrate significant transmetallation of macrocyclic agents under physiologic conditions (Coimbra et al., 2024).

Experimental studies from our group and others suggest that gadolinium-based contrast agents may trigger downstream biological effects beyond immediate direct toxicity. Tissue-retained gadolinium may promote persistent local signaling, fibrocyte recruitment, oxidative stress, chemokine activation, and pro-fibrotic pathways (Coimbra et al., 2024; DeAguero et al., 2023; Ortonne et al., 2004; Wagner et al., 2016; Drel et al., 2016; Singh et al., 2021; Do et al., 2019). More recently, we demonstrated that chelated gadolinium could dissociate under acidic conditions (Henderson et al., 2025). Additionally, we have identified gadolinium-containing nanoparticles in human tissue that may serve as a persistent source of biologic signaling after contrast exposure (DeAguero et al., 2023). Persistent urinary gadolinium likely reflects gradual mobilization of retained gadolinium-containing material, with subsequent glomerular filtration and urinary excretion; however, the potential contribution of renal tissue retention and tubular cell uptake remains incompletely understood (Darrah et al., 2009; McDonald et al., 2018; Cunningham et al., 2024; Coimbra et al., 2024; DeAguero et al., 2023; Wagner et al., 2016). Differences in gadolinium-containing nanoparticle burden may help explain variability in patient symptoms. Importantly, even macrocyclic agents have been shown to break down *in vitro* under biologically relevant conditions (Henderson et al., 2025).

In this report, we present a plausible framework rather than an established causal explanation for the patient’s gadolinium-associated symptoms. We hypothesize that our patient’s host-specific connective tissue biology, as reflected in the subsequent diagnosis of hypermobile Ehlers-Danlos syndrome (hEDS), may influence biological responses to retained gadolinium (Chiarelli et al., 2019; Reitsma et al., 2007; Merry et al., 2022; Uhl et al., 2017; Cheng et al., 2020; Proudfoot et al., 2017; Polewski et al., 2025). Given that connective tissues are recognized sites of gadolinium retention, extracellular matrix abnormalities could plausibly influence tissue persistence, redistribution, the formation of gadolinium-containing nanoparticles, or downstream signaling. However, these possibilities are considered speculative and have not been demonstrated in hEDS.

Observational studies have reported a predominance of female patients of Central and Northern European ancestry among individuals with GDD/SAGE, although the significance of this finding remains uncertain (Semelka et al., 2016). Given the patient’s European ancestry and the possibility that genetic factors may influence responses following GBCA exposure, we examined the patient’s functional genetic analysis for variants within pathways relevant to extracellular matrix biology, immune signaling, oxidative stress, detoxification, cellular repair, and neuromodulation (McDonald et al., 2018; Cunningham et al., 2024; Domingo and Semelka, 2025; Coimbra et al., 2024; Wagner et al., 2016).

Several identified variants, selected *a priori*, map to fibrosis-related and broader host-response pathways (Supplementary Table S1). To date, no genes have been definitively associated with susceptibility to NSF or with the persistent symptoms reported after gadolinium exposure, described as GDD/SAGE. Accordingly, this analysis is exploratory and hypothesis-generating, and interpretation focuses on pathway-level observations rather than the effects of individual variants. Multiple common polymorphisms affecting interconnected pathways could theoretically influence host responses to retained gadolinium.

This case highlights ongoing concerns about gadolinium-based contrast agents. Beyond the mechanistic and diagnostic uncertainties, this case underscores the practical challenges faced by patients and clinicians when causality is in question and rigorous clinical studies are lacking. The patient’s perspective illustrates the burden of persistent symptoms following gadolinium exposure and the challenges patients may face when pre-procedure education is limited, diagnostic uncertainty persists, and guidance regarding prognosis and management remains inadequate. These challenges may be further compounded by the rare reports of symptoms associated with gadolinium exposure in the scientific literature.

### Strengths

Strengths of this report include a temporal association between symptom onset and contrast exposure; prolonged urinary gadolinium excretion despite preserved renal function; serial measurements documenting delayed excretion and eventual normalization; objective documentation of dermatologic findings; multispecialty evaluations, including toxicology and robust functional genetic analyses, without identification of an alternative diagnosis; and resolution over time of symptoms except for skin changes, which showed significant but incomplete improvement.

### Limitations

Limitations include the lack of direct tissue-level confirmation of gadolinium retention, as no specific phenotype, mechanistic pathway, or validated biomarkers have been identified. Tissue gadolinium measurements were not available.

Prolonged urinary gadolinium excretion was documented and temporally associated with symptom onset; however, a causal relationship cannot be established.

Urinary creatinine concentration in the 24-h urine collections, which validates the accuracy of the collection, was not measured. The patient, however, stated that she was fastidious in following the collection directions.

A skin biopsy was not obtained during the active phase of the dermatologic findings. Subsequent dermatologic review suggested that a skin biopsy may have been helpful earlier in the course for diagnosis; however, the lesions had largely resolved, and the patient considered her health to be good by the time of consultation, limiting the expected yield for management changes.

A kidney biopsy, although potentially informative, was not performed because there was no accepted clinical indication for a native kidney biopsy in a patient with normal urinalysis, preserved kidney function, and normal serum chemistries. Consistent with current nephrology practice recommendations, the procedural risks were considered to outweigh the potential diagnostic benefit (Luciano and Moeckel, 2019).

Likely biases and confounding factors related to the patient’s independent research and self-directed interventions are also present. Although a single case cannot be generalized or establish causality, our report may help guide future hypothesis-driven research.

## Conclusion

This hypothesis-generating case is consistent with prolonged gadolinium retention and delayed elimination following administration of a newer macrocyclic gadolinium-based contrast agent. Host-specific factors may contribute to person-to-person variability in delayed gadolinium clearance and symptom expression after gadolinium exposure, even in patients without renal impairment. Although renal impairment remains the only well-established risk factor for NSF, the limited scientific literature on GDD/SAGE has left many questions unanswered (Grobner and Prischl, 2007; Marckmann et al., 2006; Semelka et al., 2016; McDonald et al., 2022; McDonald et al., 2018; Cunningham et al., 2024; Rydahl et al., 2008; Deo et al., 2007).

The coexistence of hypermobile Ehlers-Danlos syndrome in this patient raises questions regarding the potential role of connective tissue biology in those who experience gadolinium-associated symptoms (Malfait et al., 2017; Chiarelli et al., 2019; Semelka and Ramalho, 2023). Connective tissue abnormalities, autoimmune conditions, and other host factors have been proposed as potential susceptibility factors; however, these associations remain unproven and are limited by incomplete mechanistic understanding and biases inherent in self-reported registries (Burke et al., 2016; Semelka et al., 2016; Semelka and Ramalho, 2023).

Regulatory bodies and researchers agree on the need for additional research (McDonald et al., 2018; Cunningham et al., 2024; U.S. Food and Drug Administration, 2017). Until mechanisms and risk factors are better defined, clinical decision-making should continue to balance the established diagnostic benefit of MRI contrast agents against uncertain long-term risks. Future studies should identify meaningful post-exposure phenotypes beyond NSF, if any exist.

Improved mechanistic understanding may further strengthen the safety profile of these agents, including the more stable macrocyclics, and guide the development of safer MRI contrast agents.

## Data Availability

The original contributions presented in the study are included in the article/Supplementary Material, further inquiries can be directed to the corresponding authors.
