# Supplementary material for: Case Report: A hypothesis-generating case of gadolinium retention and persistent symptoms after MRI despite normal renal function in a patient with hypermobile Ehlers-Danlos syndrome
Source: Front Toxicol. 2026 Jul 16;8:1846464. doi: 10.3389/ftox.2026.1846464 (PMC13427036; doi:10.3389/ftox.2026.1846464)
Supplement: Supplementary file 1 [file Table1.docx]

**Supplementary Table S1. Genetic polymorphisms identified in biological pathways potentially relevant to host responses following gadolinium exposure.**

| **Gene** | **RS ID** | **Geno-type** | **Allele Reported** | **gnomAD Allele Frequency (%) ^†^** | **Clinical Classification** | **Functional Interpretation** |
| --- | --- | --- | --- | --- | --- | --- |
| *SPP1* | rs2853744 | GG | G | 86 | No established pathogenicity | Osteopontin expression, macrophage recruitment, and fibrosis-related signaling |
| *SPP1* | rs1126616 | TT | T | 28 | No established pathogenicity | Osteopontin expression and inflammatory signaling pathways |
| *NLRP3* | rs10754558 | GC | C | 60 | No established pathogenicity | Inflammasome activation and interleukin-1β signaling |
| *NFE2L2* | rs35652124 | TC | C | 31 | No established pathogenicity | Nuclear factor erythroid 2-related factor 2 (NRF2)-mediated antioxidant response |
| *GSTP1* | rs1695 | GG | G | 34 | Pharmacogenomic variant | Glutathione-dependent detoxification and oxidative stress response |
| *IL1B* | rs16944 | GG | G | 57 | No established pathogenicity | Interleukin-1β-mediated inflammatory signaling |
| *IL10* | rs1800896 | TC | C | 40 | No established pathogenicity | Interleukin-10-mediated anti-inflammatory immune regulation |
| *BDNF* | rs6265 | TC | T | 19 | No established pathogenicity | Neuronal plasticity, neuromodulation, and pain processing |
| *ERCC5* | rs17655 | CG | C | 24 | No established pathogenicity | Nucleotide excision repair and cellular stress response |

**Notes**

**†** Allele frequencies are from the Genome Aggregation Database (gnomAD v4.1.1), global population, combining exomes and genomes unless otherwise noted, and indicate how common each of the patient’s observed alleles is in the reference population. The allele reported in each row is the patient’s allele indicated in the “Allele Reported” column (for heterozygous genotypes, the variant/non-reference allele the patient carries). Frequencies vary across ancestral populations. Note that for rs2853744 (G) and rs16944 (G) the patient’s allele is the reference/major allele at that locus.

rs1800896 (*IL10*) and rs16944 (*IL1B*) have no exome data in gnomAD v4.1.1; their frequencies are derived from genomes only.

rs35652124, rs6265, rs17655, and rs1695 carry a gnomAD “discrepant frequencies” advisory (exome/genome differences); the combined total frequency is reported. rs1695 is multiallelic; the common G alternate allele is reported.

Variants were selected *a priori* based on reported involvement in biological pathways relevant to extracellular matrix biology, inflammation, oxidative stress, detoxification, cellular repair, and neuromodulation.

Clinical classifications reflect current population and clinical genetics databases and do not imply diagnostic, predictive, or causal relevance to NSF, GDD, SAGE, gadolinium retention, or persistent symptoms following gadolinium exposure. Functional interpretations are based on the published literature cited in Supplemental References S1–S13.

**Supplemental References**

S1. Lang F, Li Y, Yao R, Jiang M. Osteopontin in Chronic Inflammatory Diseases. Biology (Basel). 2025;14(4):428. doi:10.3390/biology14040428

S2. Jung S, Ha J, Park JH, Yoo KH. Decoding SPP1 regulation. Mol Cells. 2025;48(6):100215. doi:10.1016/j.mocell.2025.100215

S3. Shimoyama Y, Mitsuda Y, Tsuruta Y, et al. Nrf2 polymorphism in oxidative stress. Int J Med Sci. 2014;11:726–731. doi:10.7150/ijms.8590

S4. Ma Q. Nrf2 in oxidative stress. Annu Rev Pharmacol Toxicol. 2013;53:401–426. doi:10.1146/annurev-pharmtox-011112-140320

S5. Gong JY, Peng SY, Xing K, Fan L, Tan SL, Luo ZY, Yuan HY, Xu P, Luo JQ. Evaluating the role of GSTP1 genetic polymorphism (rs1695, 313A>G) as a predictor in cyclophosphamide-induced toxicities. Medicine (Baltimore). 2021 Mar 19;100(11):e24423. doi: 10.1097/MD.0000000000024423

S6. Kelley N, Jeltema D, Duan Y, He Y. NLRP3 inflammasome overview. Nat Rev Immunol. 2019;19:477–489. doi:10.1038/s41577-019-0165-0. Zhang Q, Fan HW, Zhang JZ, Wang YM, Xing HJ

S7. Dinarello CA. Interleukin‑1 in inflammatory disease. Immunity. 2011;34:831–841. doi:10.1016/j.immuni.2011.05.010

S8. Couper KN, Blount DG, Riley EM. IL‑10 immune regulation. J Immunol. 2008;180:5771–5777. doi:10.4049/jimmun

S9. Clarkson SG, Wood RD. Nucleotide excision repair polymorphisms. Mutat Res. 2005;577:195–208. doi:10.1016/j.mrrev.2005.06.003

S10. Zhao J, Chen S, Zhou H, Zhang T, Liu Y, He J, Zhu J, Ruan J. *XPG* rs17655 G>C polymorphism associated with cancer risk: evidence from 60 studies. Aging (Albany, NY). 2018 May 20;10(5):1073-1088. doi: 10.18632/aging

S11. Schärer OD. Nucleotide excision repair. Cold Spring Harb Perspect Biol. 2013;5:a012609 doi:10.1101/cshperspect.a012609

S12. Tian Y, Liu X, Jia M, Yu H, Lichtner P, Shi Y, Meng Z, Kou S, Ho IHT, Jia B, Cheng BCP, Lam CKM, Tsang S, Wong SH, Yu J, Cheng CHK, Gin T, Wu WKK, Chen Z, Chan MTV; Persistent Pain after Surgery Study Investigators. Targeted Genotyping Identifies Susceptibility Locus in Brain-derived Neurotrophic Factor Gene for Chronic Postsurgical Pain. Anesthesiology. 2018 Mar;128(3):587-597. doi: 10.1097/ALN.0000000000001977. Erratum in: Anesthesiology. 2018 May;128(5):1049. [doi:10.1097/ALN.0000000000001977](https://doi.org/10.1097/ALN.0000000000001977)

S13. Merighi A. Brain-Derived Neurotrophic Factor, Nociception, and Pain. Biomolecules. 2024 Apr 30;14(5):539. doi: 10.3390/biom14050539
